# Poor maternal nutritional status before and during pregnancy is associated with suspected child developmental delay in 2-year old Brazilian children

**DOI:** 10.1038/s41598-020-59034-y

**Published:** 2020-02-05

**Authors:** Paulo A. R. Neves, Giovanna Gatica-Domínguez, Iná S. Santos, Andréa D. Bertoldi, Marlos Domingues, Joseph Murray, Mariângela F. Silveira

**Affiliations:** 1 Postgraduate Program in Epidemiology, School of Medicine, Universidade Federal de Pelotas. Rua Marechal Deodoro, 1160, Centro 96020-220 Pelotas, Brazil; 2 Postgraduate Program in Physical Education, School of Physical Education, Universidade Federal de Pelotas. Rua Luís de Camões, 625, Três Vendas, 96055-630 Pelotas, Brazil

**Keywords:** Epidemiology, Risk factors

## Abstract

Inadequate pre-pregnancy BMI and gestational weight gain (GWG) have been associated with sub-optimal child development. We used data from the 2015 Pelotas (Brazil) Birth Cohort Study. Maternal anthropometry was extracted from antenatal/hospital records. BMI (kg/m^2^) and GWG (kg) adequacy were classified according to WHO and IOM, respectively. Development was evaluated using the INTER-NDA assessment tool for 3,776 children aged 24 months. Suspected developmental delay (SDD) was defined as <10th percentile. Associations between maternal exposures and child development were tested using linear and logistic regressions. Mediation for the association between BMI and child development through GWG was tested using G-formula. Sex differences were observed for all child development domains, except motor. Maternal pre-pregnancy underweight increased the odds of SDD in language (OR: 2.75; 95%CI: 1.30–5.80), motor (OR: 2.28; 95%CI: 1.20–4.33), and global (OR: 2.14; 95% CI: 1.05–4.33) domains for girls; among boys, excessive GWG was associated with SDD in language (OR: 1.59; 95%CI: 1.13–2.24) and cognition (OR: 1.59; 95%CI: 1.15–2.22). Total GWG suppressed the association of pre-pregnancy BMI with percentiles of global development in the entire sample. Maternal underweight and excessive GWG were negatively associated with development of girls and boys, respectively. The association of pre-pregnancy BMI with global child development was not mediated by GWG, irrespective of child’s sex.

## Introduction

Optimal child development is critical to health, and adult capacity to contribute effectively to society and the economy^1^. Yet, an estimated number of 250 million children under five years of age from low- and middle-resource settings (43%) will not achieve their full developmental potential due to social, economic, nutritional, and learning opportunity constraints^2^. A recent longitudinal multi-site study from pregnancy through childhood demonstrated that when all nutritional and health requirements are met, child development will be satisfactory and similar regardless of the geographical location^3^.

Neuroscience research has systematically shown that adverse exposures from the womb and throughout childhood, negatively affect brain development, especially for children younger than three years of age^2,4^. However, the effect of preconception and prenatal maternal malnutrition on child development is still inconsistent^2,5^. According to the Institute of Medicine (IOM), there is some evidence suggesting that a negative energy balance during pregnancy is related to impaired intellectual development in childhood^5^.

Most evidence on adverse consequences of maternal poor nutritional status, concerning pre-pregnancy body mass index (BMI) and gestational weight gain (GWG), on offspring intelligence and neurodevelopment from childhood to early adolescence, come from high-resource settings. These studies showed that both exposures present a non-linear relationship with scores for intelligence and neurodevelopment from 4 to 14 years of age, highlighting the hazardous effects of maternal pre-pregnancy underweight or overweight/obesity, as well as insufficient or excessive GWG^6–11^.

Few studies of the association of maternal anthropometry and child development have been conducted in low- and middle-income countries^12–14^. Two studies carried out in rural China found that average pre-pregnancy BMI and weekly GWG were directly associated with neuro and intellectual development measured throughout childhood; in the same study, maternal pre-pregnancy underweight was negatively associated with verbal comprehension^12,13^.

Despite the growing body of evidence, few studies investigated sex differences, with regards to child development differences between boys and girls, linked with maternal nutritional status^15,16^, finding that preconception maternal overweight and obesity was associated with lower mental and psychomotor scores only among boys at ages of 3 and 7 years in a low-income cohort in the U.S. The results were partially explained by a sex difference in growth rate in utero, which is more accelerated in boys than girls, leaving males more susceptible to adverse exposures in womb than females^16^, or due to still under investigation pathways involving placenta^15^. However, additional studies are lacking, and the mechanisms underpinning such associations remain unclear and are likely to be complex^15–17^.

Optimal child development is a key resource for societies to thrive, and it is part of the Sustainable Development Goals set for 2030^18^. As such, determining underlying drivers that might impair optimal development in childhood is essential to prevent such factors, including those that occur prenatally, burdening child development and future outcomes. Most existing studies on the effect of maternal prenatal nutrition on neurodevelopment in childhood come from high-resource settings and were carried out before the current global epidemy of obesity^6–11^. The recent findings by Widen *et al*.^15^ and Nichols *et al*.^16^ are of great importance, showing the necessity to consider sex differences when studying child development. As such, there is an urgent need for new investigations in low- and middle-income countries addressing potential drivers of impairments in childhood development. Therefore, we aimed to explore the independent associations of pre-pregnancy BMI and total GWG on different early childhood development domains (language, cognitive, motor, and global), evaluating potential differences according to child sex. Additionally, we investigated whether the association between pre-pregnancy BMI and child development was mediated by total GWG. Our general hypothesis is that both poor BMI status preconception and inadequate total GWG have negative effects on development of 2-year old Brazilian children, and also, that total GWG mediates the relationship between pre-pregnancy BMI and child neurodevelopment achievements.

## Methods

### Participants

The present prospective investigation used information from the 2015 Pelotas Birth Cohort, a population-based longitudinal study in Southern Brazil. The city has approximately 340,000 inhabitants, of whom more than 93% live in the urban area^19,20^. In 2015, when the study started, 99.9% of all births in the city occurred in hospitals. All hospital deliveries were identified (January 1^st^ to December 31^st^) through daily visits to the five hospitals with a maternity ward in the city. The newborns were evaluated, and their mothers interviewed shortly after delivery (perinatal study). Follow-up assessments took place when children were 3 and 12 months of age, through home-visits, and at 24 months of age at a research center. Further details of the study procedures can be found elsewhere^19^. The current analyses are based on information from the perinatal and 24-month assessments.

### Outcome assessment

Child development was assessed by trained examiners when children were at about 24 months of age, using the INTERGROWTH-21^st^ Neurodevelopment Assessment (INTER-NDA)^21^. This instrument uses a mixed approach to assess multiple dimensions of neurodevelopment in children aged 22–26 months. It was developed for use by non-specialists in child development in low- and high-resource settings, and it is based on an objective assessment of child performance on developmental tasks free from cultural biases^21,22^. The complete version of the package embraces four physiological and neurodevelopmental aspects of child development: vision, auditory function, sleep, and neuropsychological function^21^. The INTER-NDA takes 15–20 minutes to administer and has been widely used in different countries^21,22^. The score system of the INTER-NDA can be found detailed elsewhere^21^. The instrument was evaluated against the Bayley scale in a sub-sample of the INTERGROWTH 21st from Oxford, UK, showing good sensitivity (~70%) and specificity (~99%), as well as good agreement (interclass correlation coefficient: ~0.89)^22^. Because cognitive, language and motor domains are the neuropsychological developmental indicators most widely studied in the scientific and clinical contexts, we restricted analyses to these outcomes^23^. Each domain is scored on a 5-point scale representing the child’s performance^22^. Also, we analyzed a global score calculated as the average of the three specific neuropsychological domains and adjusted by child’s age. We dichotomized scores on each of the cognitive, language, motor, and global domains to represent below 10^th^ percentile as suspected child developmental delay, based on the entire 2015 Pelotas cohort study^24^.

### Exposures assessment

#### Pre-pregnancy nutritional status

Maternal weight at the beginning of gestation was obtained from prenatal register cards, or by maternal report at delivery if the information was not available on the card. A higher agreement between the weight reported and registered on the card was found in this population (Interclass correlation coefficient: 0.94, n = 1,406). Women’s height was measured at home during the 3-month follow-up assessment, with a portable stadiometer to the nearest 1 mm^25^. Pre-pregnancy body mass index (BMI) was calculated by dividing maternal pre-pregnancy weight by maternal squared height. We classified pre-pregnancy BMI according to WHO cut-points as follows^26^: underweight BMI < 18.5 kg/m^2^, normal weight BMI 18.5 to <25 kg/m^2^, overweight BMI 25 to <30 kg/m^2^, or obesity BMI ≥ 30 kg/m^2^.

#### Gestational weight gain

We asked mothers during the perinatal interview about their weight at the end of pregnancy, before delivery^25^. We then calculated total GWG by subtracting the final pregnancy weight from the pre-pregnancy weight. We adopted guidelines from the IOM to classify GWG in relation to pre-pregnancy BMI, in accordance with guidelines from the Ministry of Health of Brazil for antenatal care^27^. These specify that previously underweight women should gain between 12.5 to 18 kg in pregnancy, normal-weight women between 11.5 to 16 kg, overweight women between 7 to 11.5 kg, and obese women between 5 to 9 kg. Based on these weight gain ranges, we then categorized the total GWG as insufficient, adequate, or excessive^28^.

#### Covariates

The following covariates were assessed in the perinatal interview and classified as follows: maternal age (<20 years, 20 to 35 years, and ≥35 years), maternal education - years of formal schooling (≤4 years, 5 to 8 years, 9 to 11 years, ≥12 years), maternal skin color (white, black, brown/other), mother living with a partner (yes or no), maternal occupation (paid or unpaid job), parity (number of previous deliveries, including stillbirths and excluding abortions/miscarriage - 1, 2 or ≥3), and smoking during pregnancy (yes or no). Self-reported skin color definition and classification have been officially adopted in Brazil and supported by the Organized Black Movement^29^. Participant monthly family income (during the last month prior to the interview) was reported by mothers and treated as a continuous variable, then split into quintiles. The number of antenatal care visits was collected from prenatal register cards (<6, 6 to 8, ≥9). The Ministry of Health of Brazil recommends women to attend at least 6 antenatal care visits^27^.

In terms of information about the newborns, birth weight was measured within 24 hours of birth by the research team following protocols previously used in the other birth cohorts conducted in the same city^19,30^. Low birth weight was classified as weight at birth <2,500 g. The gestational age at birth was assessed by the best obstetric estimate, through available ultra-sound exams performed in one of the first two trimesters of pregnancy. When ultra-sound exams were unavailable, the self-reported date of the last menstrual period was used^30^. About 80% of the gestational age data was estimated through the best obstetric estimate in the 2015 Pelotas Birth Cohort. Prematurity was defined as birth <37 gestational weeks. At the 24-months follow-up visit, mothers were asked whether the child was still being breastfed at that time (yes or no).

### Ethical standards

Written informed consent was obtained from all mothers at the time they were invited to participate. The study was approved by the Ethics Committee of the School of Physical Education, Universidade Federal de Pelotas. All procedures were in accordance with the 1964 Declaration of Helsinki and its later amendments. Written consent was obtained from all participants or legal guardians.

### Data analysis

Analyses were restricted to singleton births. As sex differences in neurodevelopment during childhood have been previously reported^31^, we tested for an interaction between each developmental domain score, child’s sex, and the exposures. We found strong evidence for interaction in all domains, but not motor (p < 0.001). Therefore, we present results for the entire cohort, as well as stratified by child sex.

First, we compared participants followed-up at 24 months with those not assessed in relation to maternal, obstetric, and child characteristics using the chi-squared and Fisher’s exact tests. Also, we compared the characteristics of participants according to each exposure in the analytical sample. We then explored univariate associations between domains of suspected child development delay and covariates by sex of the child. The selection of covariates was done using a backward strategy, retaining in the adjusted models those associated with the outcomes at p < 0.20, as well as consideration of their relevance in the literature. Covariates chosen were: maternal age, maternal schooling, family income, maternal occupation, maternal skin color, parity, smoking in pregnancy, and gestational age at birth; all GWG models were further adjusted for the number of antenatal care visits. A conceptual framework was developed to guide analyses of the relationships between the variables under study (Supplementary Fig. 1).

Fractional polynomials were used to test departures from linearity between each of the exposures and outcomes separately. We did not find any evidence of a non-linear relationship between the variables (P > 0.05). Therefore, linear regression models were adopted, using both exposures in the continuous form and INTER-NDA domain scores as percentiles, with further adjustment for covariates. Next, crude and adjusted logistic regression models were used to analyze associations between categories of pre-pregnancy BMI and total GWG adequacy with suspected child development delay, by domains. Normal pre-pregnancy BMI and adequate GWG were used as reference categories. We ran three sets of sensitivity analyses, excluding: 1) women with pre-pregnancy BMI > 40 kg/m^2^; 2) women with any type of diabetes in pregnancy; and 3) preterm children. Missing-value categories were created to be included in the logistic adjusted models (<4% of missing).

We used G-computation to estimate the extent to which the association between continuous pre-pregnancy BMI and percentiles of child development scores were mediated by total GWG^32^. Baseline confounders included in these analyses were the covariates associated with the outcomes in the previous backward analysis, plus continued breastfeeding at 2 years. We included preterm birth and low birth weight as post confounders. To set the equations in G-computation, we used linear regression for the exposure, outcome, and mediator, and logit for the remaining equations. Bias-corrected estimates were obtained via bootstrap methods (based on 10,000 bootstrapped samples). The level of significance adopted was 5% in two-tailed tests. All analyses were performed using Stata 15.0 (Stata Corp LP, College Station, Texas, USA).

## Results

**Figure 1:**
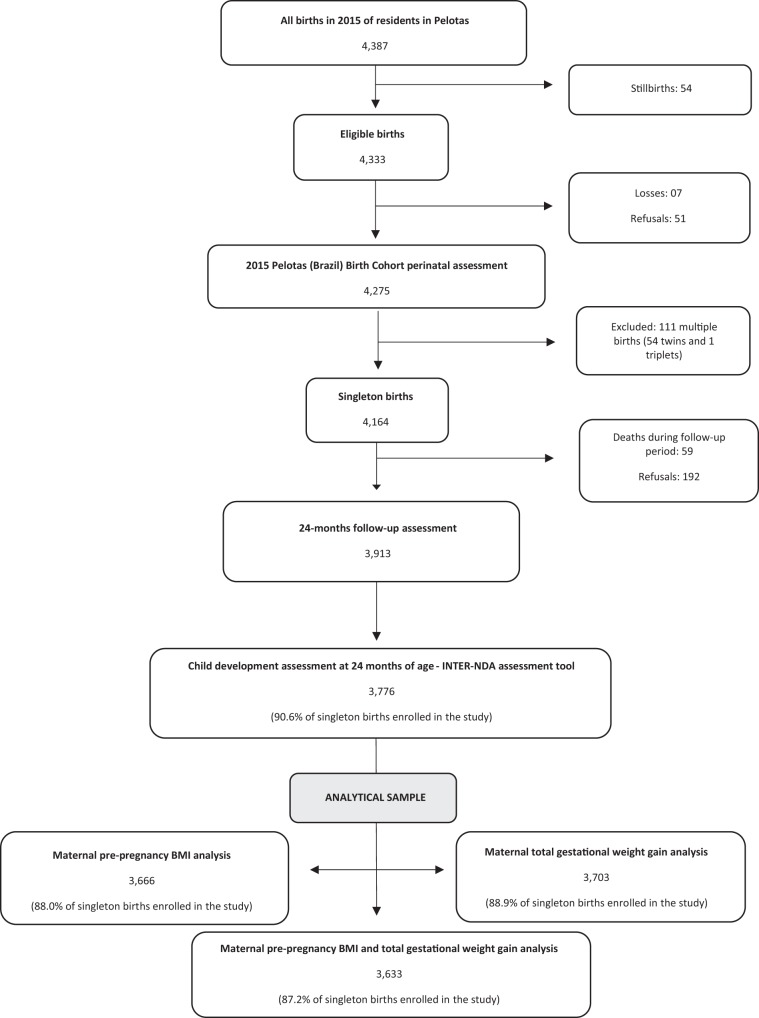
Recruitment of participants and child development assessment at 24-months follow-up in The 2015 Pelotas Birth Cohort. Adapted from Hallal *et al*.^19^.

Figure 1 shows the recruitment and follow-up flowchart of participants in the study. Of 4,333 eligible births in the reference year, 4,275 were enrolled in the perinatal study (98.7%), and 4,164 were singleton births (97.4%). Of these, 3,913 children attended the follow-up assessment at 24 months of age (94% of singleton births), and 3,776 children had their development assessed (90,6% of singleton births). Maternal information on pre-pregnancy BMI, total GWG, and both exposures by domains of development were available for 3,666 (88.0%), 3,703 (88.9%), and 3,633 (87.2%) of singleton births, respectively.

Table 1 shows the baseline characteristics of mothers and singleton children followed-up in the 24-months assessment, and comparisons between them and children unassessed on their development. With respect to maternal characteristics, less than 15% were adolescents, about 30% completed 12 or more years of schooling, most self-reported having white skin color (70.6%), and fewer more than 55% had a paid job. About 16% of mothers smoked during pregnancy and 13% did not attend at least 6 antenatal care visits (Table 1). The mean pre-pregnancy BMI and total GWG were 25.7 kg/m^2^ (Standard Deviation (SD) 5.3) and 11.9 kg (SD 6.6), respectively (data not shown in tables). In relation to pre-pregnancy BMI, almost half of the mothers were overweight (27.6%) or obese (19.5%), and only 3.8% were underweight at pregnancy onset. The frequencies of insufficient, adequate, and excessive total GWG according to IOM guidelines were 30.8%, 33.9%, and 35.3%, respectively (Table 1).

**Table 1:** Baseline characteristics of participants according to developmental assessment at 24 months in the 2015 Pelotas (Brazil) Birth Cohort (n = 3,913).

Characteristics	Children with development assessed (n = 3,776)^a^		Children with development unassessed (n = 137)		P^c^
	n^b^	Values (%)	n^b^	Values (%)	
Maternal age (years)	3,776		137		0.526
<20		548 (14.5)		22 (16.1)	
20–35		2,686 (71.1)		94 (68.6)	
≥35		542 (14.4)		21 (15.3)	
Maternal schooling (years)	3,775		137		0.115
0–4		336 (8.9)		14 (10.2)	
5–8		983 (26.0)		33 (24.1)	
9–11		1,316 (34.8)		40 (29.2)	
≥12		1,140 (30.2)		50 (36.5)	
Maternal skin color	3,770		137		0.362
White		2,660 (70.6)		97 (70.8)	
Black		608 (16.1)		18 (13.1)	
Brown or others		502 (13.3)		22 (16.1)	
Family income (quintiles)	3,774		137		0.159
Poorest		753 (20.0)		33 (24.1)	
Second		753 (20.0)		30 (21.9)	
Third		763 (20.2)		21 (15.3)	
Fourth		777 (20.6)		22 (16.1)	
Richest		728 (19.2)		31 (22.6)	
Mother living with a partner	3,775		137		0.667
Yes		3,247 (86.0)		116 (84.7)	
No		528 (14.0)		21 (15.3)	
Maternal occupation	3,776		137		0.03
Paid job		2,121 (56.2)		64 (46.7)	
Unpaid job		1,655 (43.8)		73 (53.7)	
Parity	3,774		137		0.214
1		1,862 (49.3)		75 (54.7)	
2		1,174 (31.1)		37 (27.0)	
≥3		738 (19.6)		25 (18.3)	
Smoking in pregnancy	3,773		137		0.661
Yes		611 (16.2)		24 (17.5)	
No		3,162 (83.8)		113 (82.5)	
Number of antenatal care visits	3,697		132		0.382
<6		486 (13.2)		19 (14.4)	
6–8		1,331 (36.0)		51 (38.6)	
≥9		1,880 (50.8)		62 (47.0)	
Pre-pregnancy BMI (kg/m^2^)	3,666		134		0.154
<18.5		144 (3.9)		2 (1.5)	
18.5–24.9		1,797 (49.0)		69 (51.5)	
25.0–29.9		1,009 (27.5)		40 (29.8)	
≥30.0		716 (19.5)		23 (17.2)	
Total gestational weight gain (kg)^d^	3,633		131		0.04
Insufficient		1,128 (31.0)		30 (22.9)	
Adequate		1,235 (34.0)		43 (32.8)	
Excessive		1,270 (35.0)		58 (44.3)	
Type of delivery	3,775		137		0.374
Vaginal		1,356 (35.9)		44 (32.1)	
Cesarean section		2,419 (64.1)		93 (67.9)	
Birth weight (grams)	3,774		136		0.44
<2,500		295 (7.8)		8 (5.9)	
2,500–3,500		2,444 (64.8)		89 (65.4)	
≥3500		1,035 (27.4)		39 (28.7)	
Preterm birth (<37 weeks gestation)	3,776		137		0.323
Yes		498 (13.2)		14 (10.2)	
No		3,278 (86.8)		123 (89.8)	
Sex of the child	3,776		137		0.141
Boy		1,924 (50.9)		61 (44.5)	
Gi rl		1,852 (49.1)		76 (55.5)	

^a^Only singleton births; ^b^Totals differ due to missing values; ^c^Pearson chi-squared or Fisher’s exact tests for comparison between children’s characteristics followed-up at 24-months of age and those lost to follow-up; ^d^According to Institute of Medicine guidelines, 2009^5^.

Regarding child characteristics, 13.8% were born prematurely and 8.3% weighed less than 2,500 g at birth. The proportion of girls in the sample was 49.2% (Table 1). More than one-quarter of children (27.1%) were still being breastfed at 24 months of age (data not shown in tables). Children who were followed-up at the 24-months assessment only differed to those whose development was not assessed with respect to maternal total GWG and occupation; mothers of children with unassessed development were more likely to have inadequate total GWG (insufficient or excessive), and less likely to have a paid occupation (Table 1).

The prevalence of suspected child neurodevelopmental delay at 24 months of age for each domain were as follows: 10.2% for global; 10% for language, 11.4% for cognitive, and 10% for motor domain. Girls were significantly less likely to have suspected developmental delay in comparison to boys in all domains, except motor, which was similar for both sexes (Supplementary Table S1). We did not observe differences in characteristics of participants in relation to exposures studied in the analytical samples (Supplementary Table S2). Suspected child development delay was associated with the number of antenatal care visits, birth weight, and prematurity among boys and with maternal schooling, family income, maternal occupation, parity, number of antenatal care visits, newborn birth weight, and prematurity among girls (Supplementary Tables S3 and S4).

In the crude regression model, only total GWG was associated with the percentiles of language and cognitive scores among girls (Supplementary Table S5). However, after controlling for covariates the association disappeared (Table 2). On the other hand, for each kilogram-unit increment in total GWG there was a reduction of 0.007 percentiles in the INTER-NDA scores in the global (95% CI: −0.013 to −0.000), language (95% CI: −0.014 to −0.000), and cognitive (95% CI: −0.014 to −0.000) domains, respectively, among boys in the adjusted regression model (Table 2).

**Table 2:** Adjusted linear regression models of the associations between pre-pregnancy body mass index and total gestational weight gain with INTER-NDA score percentiles by domain at 24-month follow-up assessment in the 2015 Pelotas (Brazil) Birth Cohort, stratified by child’s sex.

	Global	Language	Cognitive	Motor
	β (95% CI)	β (95% CI)	β (95% CI)	β (95% CI)
**Total sample**				
Pre-pregnancy BMI (kg/m^2^)^a,b^	0.000 (−0.005; 0.007)	0.000 (−0.005; 0.006)	0.001 (−0.004; 0.007)	0.005 (−0.001; 0.011)
Total gestational weight gain (kg)^c,d^	−0.002 (−0.007; 0.002)	−0.001 (−0.006; 0.003)	−0.001 (−0.006; 0.003)	−0.004 (−0.01; 0.000)
**Boys**				
Pre-pregnancy BMI (kg/m^2^)^a,b^	0.002 (−0.005; 0.011)	0.003 (−0.005; 0.011)	0.004 (−0.003; 0.012)	0.003 (−0.004; 0.011)
Total gestational weight gain (kg)^c,d^	−0.007 (−0.013; −0.000)	−0.007 (−0.014; −0.000)	−0.007 (−0.014; −0.000)	−0.006 (−0.013; 0.000)
**Girls**				
Pre-pregnancy BMI (kg/m^2^)^a,b^	0.000 (−0.008; 0.010)	−0.000 (−0.009; 0.008)	0.001 (−0.007; 0.010)	0.006 (−0.003; 0.015)
Total gestational weight gain (kg)^c,d^	0.001 (−0.005; 0.008)	0.004 (−0.002; 0.011)	0.004 (−0.002; 0.010)	−0.002 (−0.010; 0.004)

INTER-NDA – Intergrowth-21st Neurodevelopment Assessment. BMI – body mass index. 95% CI − 95% confidence interval.

^a^n = 3,666. ^b^Model adjusted for maternal age, maternal schooling, family income, maternal occupation, maternal skin color, parity, smoking in pregnancy, gestational age at birth.

^c^n = 3,703. ^d^Same model as for pre-pregnancy BMI plus adjustment for the number of antenatal care appointments.

Unadjusted logistic regression showed that excessive total GWG was associated with higher odds of suspected delay in language and cognition domains among boys. For girls, maternal preconception underweight increased the odds for suspected delays in global, language and motor development (Supplementary Table 6). Table 3 shows results for adjusted analysis. Insufficient GWG was associated with lower odds for motor delay in the entire sample (OR: 0.73, 95% CI: 0.55 to 0.97). Among boys, excessive GWG increased the odds of suspected delay in language (OR: 1.59, 95% CI: 1.13 to 2.24) and cognitive (OR: 1.59, 95% CI: 1.15 to 2.22) domains. Girls whose mothers were underweight before pregnancy presented two times higher odds of suspected delay in global (OR: 2.14, 95% CI: 1.05 to 4.33), language (2.75, 95% CI: 1.30 to 5.80) and motor (OR: 2.28, 95% CI: 1.20 to 4.33) domains.

**Table 3:** Adjusted associations between pre-pregnancy body mass index and adherence to IOM total gestational weight gain recommendations with suspected child developmental delays^a^ at 24-month follow-up assessment in the 2015 Pelotas (Brazil) Birth Cohort, stratified by child’s sex (n = 3,913).

	Global	Language	Cognitive	Motor
	OR (95% CI)	OR (95% CI)	OR (95% CI)	OR (95% CI)
**Total sample**				
**Pre-pregnancy BMI (kg/m**^**2**^**)**^**b**^				
<18.5	1.33 (0.79–2.25)	1.08 (0.61–1.90)	0.94 (0.54–1.63)	1.48 (0.89–2.44)
18.5–24.9	Reference	Reference	Reference	Reference
25.0–29.9	1.05 (0.81–1.37)	1.04 (0.80–1.35)	0.98 (0.76–1.25)	1.06 (0.81–1.37)
≥30.0	0.86 (0.63–1.17)	0.92 (0.68–1.25)	0.83 (0.62–1.11)	0.87 (0.64–1.19)
**Total gestational weight gain (kg)**^**c,d**^				
Insufficient	0.85 (0.64–1.13)	0.98 (0.74–1.31)	1.04 (0.79–1.35)	0.73 (0.55–0.97)
Adequate	Reference	Reference	Reference	Reference
Excessive	1.05 (0.81–1.36)	1.26 (0.97–1.65)	1.28 (0.99–1.65)	1.02 (0.79–1.32)
**Boys**				
**Pre-pregnancy BMI (kg/m**^**2**^**)**^**b**^				
<18.5	0.78 (0.34–1.78)	0.46 (0.18–1.17)	0.50 (0.21–1.19)	0.84 (0.35–2.02)
18.5–24.9	Reference	Reference	Reference	Reference
25.0–29.9	0.96 (0.68–1.35)	0.93 (0.67–1.30)	0.91 (0.67–1.25)	0.91 (0.62–1.32)
≥30.0	0.74 (0.50–1.10)	0.75 (0.52–1.10)	0.68 (0.47–1.00)	0.95 (0.63–1.44)
**Total gestational weight gain (kg)**^**c,d**^				
Insufficient	0.88 (0.60–1.28)	1.12 (0.77–1.62)	1.22 (0.86–1.73)	0.69 (0.45–1.06)
Adequate	Reference	Reference	Reference	Reference
Excessive	1.27 (0.90–1.79)	1.59 (1.13–2.24)	1.59 (1.15–2.22)	1.28 (0.89–1.84)
**Girls**				
**Pre-pregnancy BMI (kg/m**^**2**^**)**^**a**^				
<18.5	2.14 (1.05–4.33)	2.75 (1.30–5.80)	1.88 (0.90–3.90)	2.28 (1.20–4.33)
18.5–24.9	Reference	Reference	Reference	Reference
25.0–29.9	1.19 (0.79–1.79)	1.21 (0.77–1.89)	1.06 (0.70–1.60)	1.23 (0.86–1.77)
≥30.0	0.98 (0.60–1.61)	1.16 (0.70–1.94)	1.02 (0.64–1.64)	0.77 (0.47–1.24)
**Total gestational weight gain (kg)**^**b,c**^				
Insufficient	0.84 (0.55–1.28)	0.84 (0.54–1.32)	0.84 (0.56–1.28)	0.76 (0.52–1.12)
Adequate	Reference	Reference	Reference	Reference
Excessive	0.83 (0.54–1.27)	0.87 (0.55–1.37)	0.93 (0.62–1.41)	0.82 (0.56–1.19)

IOM – Institute of Medicine. BMI – body mass index. OR -odds ratio. 95% CI − 95% confidence interval.

^a^Suspected child development delay defined as scores of each domain below 10^th^ percentile, based on the entire 2015 Pelotas Birth Cohort.

^b^Model adjusted for maternal age, maternal schooling, family income, maternal occupation, maternal skin color, parity, smoking in pregnancy, gestational age at birth.

^c^Same model as for pre-pregnancy BMI plus adjustment for the number of antenatal care appointments.

^d^According to Institute of Medicine guidelines, 2009^5^.

In the sensitivity analyses, the results were not affected either in magnitude or direction after restricting to women with BMI < 40 kg/m^2^ or non-diabetic in the linear and logistic models. However, when restricting analyses for full-term children, only suspected delay in language domain among girls was associated with pre-pregnancy underweight (OR: 2.54, 95% CI: 1.12 to 5.77) (data not shown in tables).

Supplementary Table S7 presents the results of the mediation analysis. Twelve tests were performed: four developmental outcome domains each tested on the entire sample, for boys, and for girls. We observed suppressing mediation on the relationship between pre-pregnancy BMI and percentiles of child development through total GWG only for the global domain in the whole cohort (indirect effect: −0.057, 95% CI: −0.101 to −0.013), suggesting that the effects of pre-pregnancy BMI on global childhood developmental domain probably occur through other paths than by total GWG. No similar results were found for the other domains or stratified by child sex.

## Discussion

Our results add to the current evidence of the importance of maternal nutritional status before and during pregnancy for child development, highlighting the first 1,000 days as critical for improved lifetime achievements^33,34^. In a large prospective cohort, we showed that maternal BMI before gestation and total GWG act distinctly on the neurodevelopment of boys and girls. Pre-pregnancy maternal underweight more than doubled the odds of suspected delay in language, motor, and global domains for girls, whereas boys whose mothers had excessive weight gain in pregnancy than recommended by IOM had nearly 60% higher odds of language and cognitive developmental delays. There was little evidence of mediation in our analysis^35^, but total GWG suppressed the effect of pre-pregnancy BMI on percentiles scores of global development. The prevalence of the outcomes was higher among boys compared to girls.

Previous studies conducted in high-income countries have reported an inverse relationship between preconception maternal BMI and GWG with lower intellectual development in childhood^8–10^, with some studies showing an inverted U-shaped association^6,7^. Data from the U.S. Collaborative Perinatal Project, which enrolled more than 30,000 children, suggested that offspring born to normal-weight mothers appear to have higher scores for intelligence quotient and that low and high GWG acted detrimentally on cognition in children aged 4 to 7 years, especially if obese women gained excessive weight in pregnancy^6,7^. Another U.S. cohort found that 9-year-old children of women either overweight or obese onset in pregnancy scored significantly lower on vocabulary tests compared to children of normal weight mothers. In the same study, GWG was not associated with child vocabulary^10^. In a cohort study of non-obese Scandinavian women, no differences in components of intelligence quotient among 5-years-old children were observed by GWG adequacy in the 2^nd^ and 3^rd^ trimester of gestation^11^. Offspring academic achievement scores in math, reading, and spelling at 6, 10, and 14 years were inversely associated with pre-pregnancy BMI > 22 kg/m^2^ and high GWG in a cohort in Pennsylvania, U.S.^9^. Associations between pre-pregnancy underweight and developmental impairments and intelligence were not common, presumably because low maternal weight is not a cause of concern in these populations^36^.

Evidence from low and middle-income settings has highlighted deleterious effects associated with poor maternal nutritional status on child cognitive and mental development, especially maternal underweight^12,13^. Two Chinese studies with rural children found harmful associations between pre-pregnancy maternal underweight and cognitive development of children at 3–24 months and significantly fewer scores in verbal comprehension tests among early-school children^12,13^. Weekly GWG was positively associated with mental development only when children were 3 months old; no effects for older children were reported, suggesting that the effects of GWG might decline or disappear with age^12,13^. Yet, bias could be introduced in these results as no trimester-specific weekly GWG was examined, considering that weight gain progressively increases by gestational age. Importantly, the occurrence of maternal underweight in both surveyed populations was relatively high (about 16%)^12,13^.

Our results are in agreement with previous literature, showing that children with lower achievements in tests for intelligence and development are more likely to have been born to women with poor nutritional status before and during gestation. Similarly to our findings, Hinkle *et al*.^37^ reported an increased risk of suspected child development delay in 2-year old children born to either underweight (RR: 1.36, 95% CI: 1.04 to 1.78) or severely obese (RR: 1.38, 95% CI: 1.03 to 1.84) mothers at the beginning of pregnancy, using the Bayley-II scale to assess development in children. All analyses were adjusted by sex of the child, but stratified results were not shown^37^. However, in our study, associations between low maternal weight, and delays in language, motor, and global domains were only seen for girls. Regarding GWG, our linear models showed an inverse association between total GWG and percentile scores of global, language, and cognitive domains among boys, which are in agreement with another of our findings that excessive total GWG posed as a threat to suspected developmental delay among boys. Surprisingly, an unexpected protection effect was observed for excessive total GWG on suspected motor delay in the whole cohort, for which we could not found a plausible explanation. Conflicting evidence concerns the effect of extremes of total GWG on offspring’s brain development, and still needs further investigation as some studies fail in showing negative associations for cognition and intelligence^10,13^. These results might indicate that poor maternal nutritional status affects child development through etiological paths that have not yet been identified.

Some hypotheses have been suggested to explain our findings, considering the crucial role that nutrition has on early stages of life development, beginning in conception^38,39^. Throughout gestation, fetus brain and central nervous system growth and development occur rapidly, with high neuron proliferation, synapse formation, axon growth, and myelination^39^. This critical period is sensitive to adverse nutritional deprivation and excess, likely leading to poor foundation of brain structures, affecting the development of cognitive, motor, and emotional skills in childhood^39^. Animal models have demonstrated associations between caloric-protein shortage in pregnancy and abnormal brain development in rats, which depended on the time of onset and duration of the privation^38^. Similarly, maternal overweight and obesity may alter brain development, consequence of a poor-quality diet and unsatisfactory dietary behaviors^7,8,40^. Inflammatory and hormonal aspects related to excessive maternal weight, such as those involving leptin, and insulin disruption may lead to altered neuronal proliferation and formation, as evidenced in experimental studies with rodents^8,41^.

Our analysis failed in presenting direct mediation for the pre-pregnancy BMI relationship through total GWG in the full sample (boys and girls); in fact, the hypothesized mediator suppressed this association only for the global domain. However, no other test (for the entire sample, boys, or girls) showed evidence of mediation, meaning caution is required in interpreting this finding. We included low birth weight and prematurity as post confounders in our mediation, which might have attenuated or eliminated any effect of the exposure and mediator on the outcome. After restricting the analyses to term children, only suspected language delay among girls remained associated with preconception underweight. Analogously, Hinkle *et al*.^37^ described that after limiting analyses to full-term children, only severe obesity remained associated with poor mental development in 2-years old children in the U.S. Malnutrition in pregnancy is one of the causes of intrauterine growth restriction, low birth weight, and prematurity, which are well-known causes of abnormal development in childhood^2,42^, and may represent intermediate factors in a complex causal path between maternal malnutrition and impaired child development, with multiple routes. However, as the lower and higher categories of pre-pregnancy BMI remained associated with poor cognition in children in our study and in the U.S.^37^ even after removing preterm children from the analyses, other mechanisms related to the unbalanced nutrition in pregnancy (micronutrient deficiencies, caloric-protein disruption, obesity-related inflammatory status, placental transport disruption, specific dietary patterns) are likely to affect brain development and growth in the womb^15,40–44^. The relationship between pre-pregnancy BMI and GWG on child development is challenging to untangle; conflicting evidence describes incremental deleterious effects of excessive GWG for women who were obese pre-pregnancy^7^, as well as no modification for the effect of GWG after adjustment for pre-pregnancy BMI^8^. Yet, these studies did not use appropriate methods of testing mediation to reach these conclusions.

Some trials found that nutrition-specific interventions effectively improved development in children mostly born to underweight women^14,45,46^. A large randomized controlled trial in Bangladesh evaluating the effect of multiple micronutrients and food supplementation in early- and mid-pregnancy on problem-solving tests in childhood, evidenced significant benefits for infant development at age seven months among low BMI mothers receiving the food supplementation compared to usual-food group^14^. Results from a clinical trial conducted in Guatemala in which pregnant women and their children up to 7 years were provided with a high protein milk-based energy drink compared to low protein energy drink group showed that children from the intervention group scored more points on intelligence quotient (among women) and had increased wages (among men) in adulthood^45,46^.

An unusual aspect of our study was exploring differences between boys and girls in developmental achievements in relation to maternal nutritional status. Previously, Nichols *et al*.^16^ also reported on sex-differences in child development according to maternal nutrition, as boys of obese women at preconception scored significantly lower on psychomotor tests compared to those of normal weight mothers; similar results for girls were not found. Similarly, Widen *et al*.^15^ found that only boys born to overweight or obese women at preconception scored meaningfully lower in intelligence quotient test at 7 years in a low-income birth cohort in the U.S. By contrast, we found that development in boys was affected by excessive total GWG, whereas girls were affected by pre-pregnancy underweight. The divergence between the findings might be because of different social contexts of the populations, as well as differences in other individual factors, like the home environment, parental stimulation, and maternal intelligence quotient, which were not accounted for in our analysis. It is important to note that in utero growth and brain development occur differently between boys and girls^47^. Boys might be more susceptible to adverse exposures during pregnancy due to their faster growth rate, as well as their slower maturation than girls, which make them more vulnerable^17,47,48^. Additionally, sex-specific differences in brain structure have been described, some related to different aspects of development (cognitive vs. motor, for instance)^31^. Moreover, harmful exposures in pregnancy, such as alcohol and tobacco, were found to impact development differently by sex of the child^17^. Overall, such factors might help to explain sex differences in certain developmental domains observed in our study and elsewhere^15,16^, but the causes are unclear. There is a growing body of evidence trying to establish pathways that demonstrate why boys are more vulnerable to some exposures than girls^16,17^, however, mechanisms related to female brain development seem to be understudied. Therefore, it is essential to consider sex differences when analyzing the detrimental effects of antenatal exposures on neurodevelopment^17,31^. Such variation in the effects on child development is important, especially considering the increasing rates of obesity among women worldwide, and the persistent high to moderate magnitude of underweight in low resource settings, detrimentally affecting the in-utero environment^36^.

There are limitations to this study that need to be considered: (1) data on weekly GWG was lacking, precluding trimester-specific analysis for this exposure; (2) we relied on self-reported information of maternal weight at the beginning and the end of pregnancy if data from antenatal registers were lacking; however, there was high agreement between reported and registered preconception weight in our study, when both were available. Notably, a high agreement between self-reported and measured weights were systematically described^49^, as well as found in a large Brazilian cohort study^50^; (3) although a high agreement between the INTER-NDA and the Bayley scale was described in Oxford, UK^22^, this agreement might differ in other locations. Nonetheless, the INTER-NDA was created to be free from cultural-biases, useful in different contexts in high and low resource settings, and performed by non-specialists, therefore, reducing validation-related issues in our study; (4) we did not account for the home environment and maternal intelligence quotient as potential determinants of optimal childhood development in the adjusted analyses, as those were unavailable. Conversely, our study has several strengths: (1) the prospective longitudinal design allowed us to use properly time-ordered data in the relationship studied; (2) we relied upon information of a birth cohort conducted under rigorous field procedures with reliable data; (3) we used a novel international-standardized tool to assess child development, allowing comparison with studies of populations in high- and middle/low-income countries; (4) we used adequate methods to study mediation, accounting for post-confounders in the causal mediation path.

In conclusion, poor maternal nutritional status before and during pregnancy was associated with suspected neurodevelopmental delays in 24-month-old children in a population-based study in Brazil, with clear evidence of sex-difference in the associations. Language, motor and global development among girls was affected by pre-pregnancy underweight, as excessive total weight gain during gestation affected detrimentally language and cognitive development among boys. Mediation analysis suggests that the association of pre-pregnancy BMI with global child development at 2 years does not directly pass through total GWG, irrespective of the sex of the child. Our results emphasize the importance of optimal nutritional maternal status that needs to be addressed during antenatal care to provide the conditions for optimal child development. Future investigations stratified by sex of the child are encouraged to disentangle the intricate association of maternal BMI and GWG with neurodevelopment of children.

## Supplementary information

Supplementary information.

## Data Availability

The dataset will be made available upon request to the correspondent author.

## References

[1] Shonkoff JP (2012). The lifelong effects of early childhood adversity and toxic stress. Pediatrics..

[2] Black MM (2017). Early childhood development coming of age: science through the life course. Lancet..

[3] Villar J (2019). Neurodevelopmental milestones and associated behaviours are similar among healthy children across diverse geographical locations. Nat. Commun..

[4] Dua T (2016). Global research priorities to accelerate early child development in the sustainable development era. Lancet Glob. Health..

[5] Institute of Medicine. Weight gain during pregnancy: reexamining the guidelines. (IOM, 2009). 20669500

[6] Keim SA, Pruitt NT (2012). Gestational weight gain and child cognitive development. Int. J. Epidemiol..

[7] Huang L (2014). Maternal prepregnancy obesity and childneurodevelopment in the Collaborative Perinatal Project. Int. J. Epidemiol..

[8] Pugh SJ (2015). Maternal obesity and excessive gestational weight gain are associated with components of child cognition. J. Nutr..

[9] Pugh SJ (2016). Child academic achievement in association with pre-pregnancy obesity and gestational weight gain. J. Epidemiol. Community Health..

[10] Widen Elizabeth Marie, Kahn Linda Gross, Cirillo Piera, Cohn Barbara, Kezios Katrina Lynn, Factor-Litvak Pam (2017). Prepregnancy overweight and obesity are associated with impaired child neurodevelopment. Maternal & Child Nutrition.

[11] Hinkle SN, Albert PS, Sjaarda LA, Grewal J, Grantz KL (2016). Trajectories of maternal gestational weight gain and child cognition assessed at 5 years of age in a prospective cohort study. J. Epidemiol. Community Health..

[12] Li C (2019). Effect of maternal pre-pregnancy BMI and weekly gestational weight gain on the development of infants. Nutr. J..

[13] Li C (2018). Effect of maternal pre-pregnancy underweight and average gestational weight gain on physical growth and intellectual development of early school-aged children. Sci. Rep..

[14] Tofail F (2008). Effects of prenatal food and micronutrient supplementation on infant development: a randomized trial from the maternal and infant nutrition interventions, Matlab (MIMIMat) study. Am. J. Clin. Nutr..

[15] Widen EM (2019). Prepregnancy obesity is associated with cognitive outcomes in boys in a low-income, multiethnic birth cohort. BMC Pediatr..

[16] Nichols Amy R., Rundle Andrew G., Factor-Litvak Pam, Insel Beverly J., Hoepner Lori, Rauh Virginia, Perera Frederica, Widen Elizabeth M. (2019). Prepregnancy obesity is associated with lower psychomotor development scores in boys at age 3 in a low-income, minority birth cohort. Journal of Developmental Origins of Health and Disease.

[17] DiPietro JA, Voegtline KM (2017). The gestational foundation of sex differences in development and vulnerability. Neuroscience..

[18] United Nation Children’s Fund. Are we on track to achieve the SDGs for children? The situation in 2019 (UNICEF, 2019).

[19] Hallal PC (2018). Cohort Profile: The 2015 Pelotas (Brazil) Birth Cohort Study. Int. J. Epidemiol..

[20] Instituto Brasileiro de Geografia e Estatística (IBGE). Cidades. https://cidades.ibge.gov.br/brasil/rs/pelotas/panorama (2019).

[21] Fernandes M (2014). The INTERGROWTH-21st project neurodevelopment package: A novel method for the multi-dimensional assessment of neurodevelopment in pre-school age children. PLoS One.

[22] Murray E (2018). Evaluation of the INTERGROWTH-21st Neurodevelopment Assessment (INTER-NDA) in 2 year-old children. PLoS One..

[23] Fischer VJ, Morris J, Martines J (2014). Developmental screening tools: feasibility of use at Primary Health Care level in low- and middle-income settings. J. Heal. Popul. Nutr..

[24] Barros AJ, Matijasevich A, Santos IS, Halpern R (2010). Child development in a birth cohort: effect of child stimulation is stronger in less educated mothers. Int. J. Epidemiol..

[25] Horta BL (2019). Maternal anthropometry: Trends and inequalities in four population-based birth cohorts in Pelotas, Brazil, 1982-2015. Int. J. Epidemiol..

[26] Physical status: the use and interpretation of anthropometry (World Health Organization, Geneva, Switzerland, 1995).

[27] Primary Heath Care Series, number 32 – Antenatal care for low risk women [Caderno de Atenção Básica, n° 32 - Atenção ao pré-natal de baixo risco] (Ministério da Saúde, Brasília, Brazil, 2013).

[28] Institute of Medicine. Implementing guidelines on weight gain and pregnancy (IOM, 2013).

[29] Petruccelli, J.L. & Saboia, A.L. *Ethno-racial characteristics of the population. Classification and identities*, http://servicodados.ibge.gov.br/Download/Download.ashx?http=1&u=biblioteca.ibge.gov.br/visualizacao/livros/liv49891.pdf (2013).

[30] Silveira MF (2019). Low birthweight and preterm birth: Trends and inequalities in four population-based birth cohorts in Pelotas, Brazil, 1982-2015. Int. J. Epidemiol..

[31] Ruigrok ANV (2014). A meta-analysis of sex differences in human brain structure. Neurosci. Biobehav. Rev..

[32] Daniel RM, Stavola. BL, Cousens SN (2011). gformula: Estimating causal effects in the presence of time-varying confounding or mediation using the g-computation formula. Stata J..

[33] Black RE (2013). Maternal and child undernutrition and overweight in low-income and middle-income countries. Lancet..

[34] Stephenson J (2018). Before the beginning: nutrition and lifestyle in the preconception period and its importance for future health. Lancet..

[35] MacKinnon DP, Fairchild AJ, Fritz MS (2007). Mediation Analysis. Annu. Rev. Psychol..

[36] NCD Risk Factor Collaboration (2017). Worldwide trends in body-mass index, underweight, overweight, and obesity from 1975 to 2016: a pooled analysis of 2416 population-based measurement studies in 128.9 million children, adolescents, and adults. Lancet..

[37] Hinkle SN (2012). Associations between maternal prepregnancy body mass index and child neurodevelopment at 2 years of age. Int. J. Obes..

[38] Alamy M, Bengelloun WA (2012). Malnutrition and brain development: An analysis of the effects of inadequate diet during different stages of life in rat. Neurosci. Biobehav. Rev..

[39] Prado EL, Dewey KG (2014). Nutrition and brain development in early life. Nutr. Rev..

[40] Georgieff MK (2007). Nutrition and the developing brain: nutrient priorities and measurement. Am. J. Clin. Nutr..

[41] Tozuka Y (2010). Maternal obesity impairs hippocampal BDNF production and spatial learning performance in young mouse offspring. Neurochem. Int..

[42] Britto PR (2017). Nurturing care: promoting early childhood development. Lancet..

[43] Segovia SA, Vickers MH, Reynolds CM (2017). The impact of maternal obesity on inflammatory processes and consequences for later offspring health outcomes. J. Dev. Orig. Health Dis..

[44] Sullivan EL, Nousen EK, Chamlou KA (2014). Maternal high fat diet consumption during the perinatal period programs offspring behavior. Physiol. Behav..

[45] Hoddinott J, Maluccio JA, Behrman JR, Flores R, Martorell R (2008). Effect of a nutrition intervention during early childhood on economic productivity in Guatemalan adults. Lancet..

[46] Li H, Barnhart HX, Stein AD, Martorell R (2004). Effects of early childhood supplementation on the educational achievement of women. Pediatrics..

[47] Misra DP, Salafia CM, Miller RK, Charles AK (2009). Non-linear and gender-specific relationships among placental growth measures and the fetoplacental weight ratio. Placenta..

[48] Barker DJP, Thornburg KL, Osmond C, Kajantie E, Eriksson JG (2010). Beyond birthweight: The maternal and placental origins of chronic disease. J. Dev. Orig. Health Dis..

[49] Headen I, Cohen AK, Mujahid M, Abrams B (2017). The accuracy of self-reported pregnancy related weight: a systematic review. Obes. Rev..

[50] Araújo RGPS, Gama SGN, Barros DC, Saunders C, Mattos IE (2017). Validity of self-reported weight, height, and BMI in mothers of the research Birth in Brazil. Rev. Saude Publica..

