# Supplementary material for: Poor maternal nutritional status before and during pregnancy is associated with suspected child developmental delay in 2-year old Brazilian children
Source: Sci Rep. 2020 Feb 5;10:1851. doi: 10.1038/s41598-020-59034-y (PMC7002477; doi:10.1038/s41598-020-59034-y)
Supplement: Supplementary file 1 — Supplementary information. [file 41598_2020_59034_MOESM1_ESM.docx]

# **Poor maternal nutritional status before and during pregnancy is associated with suspected child developmental delay in 2-year old Brazilian children**

Paulo A R Neves, Giovanna Gatica-Domínguez, Iná S Santos, Andréa D Bertoldi, Marlos Domingues, Joseph Murray, Mariângela F Silveira

Figure S1. Proposed diagram of the association between maternal nutrition exposures^a^ with child development at 2 years.

Pre-pregnancy BMI

SES and antenatal characteristics

Child development at 2 years

BF at 2 years

GA at birth

BW

Total GWG

^a^Pre-pregnancy BMI (body mass index) and total GWG (gestational weight gain).

SES – socioeconomic characteristics; GA – gestational age; BW – birth weight; BF - breastfeeding

Table S1. Prevalence of suspected developmental delay^a^ at 24 months of age by sex of the child in the 2015 Pelotas (Brazil) Birth Cohort.

| Domains of development | Boys (n= 1,924) |  | Girls (n= 1,852) | P |
| --- | --- | --- | --- | --- |
|  | Prevalence (95% CI) |  | Prevalence (95% CI) |  |
| Global | 12.0 (10.6; 13.5) |  | 8.1 (7.0; 9.5) | 0.0001 |
| Language | 12.7 (11.2; 14.2) |  | 7.1 (6.0; 8.4) | <0.0001 |
| Cognitive | 14.1 (12.6; 15.7) |  | 8.6 (7.4; 10.0) | <0.0001 |
| Motor | 9.6 (8.4; 11.1) |  | 10.2 (8.9; 11.7) | 0.543 |

^a^Suspected child development delay defined as scores of each domain below 10^th^ percentile, based on the entire 2015 Pelotas (Brazil) Birth Cohort.

Table S2. Baseline characteristics of participants according to exposures investigated in the 2015 Pelotas (Brazil) Birth Cohort.

| Characteristics | Participants with pre-pregnancy BMI available  (n= 3,666)^a,c^ | |  | Participants with total GWG available  (n= 3,703)^a,c^ | |  | Participants with both exposures available  (n= 3,633)^a,c^ | |
| --- | --- | --- | --- | --- | --- | --- | --- | --- |
|  | n^b^ | Values (%) |  | n^b^ | Values (%) |  | n^b^ | Values (%) |
| Maternal age (years) | 3,666 |  |  | 3,703 |  |  | 3,633 |  |
| < 20 |  | 512 (14.0) |  |  | 530 (14.3) |  |  | 504 (13.9) |
| 20-35 |  | 2,622 (71.5) |  |  | 2,645 (71.4) |  |  | 2,604 (71.7) |
| ≥ 35 |  | 532 (14.5) |  |  | 528 (14.3) |  |  | 525 (14.4) |
| Maternal schooling (years) | 3,666 |  |  | 3,703 |  |  | 3,633 |  |
| 0-4 |  | 307 (8.4) |  |  | 315 (8.5) |  |  | 298 (8.2) |
| 5-8 |  | 939 (25.6) |  |  | 957 (25.8) |  |  | 929 (25.6) |
| 9-11 |  | 1,286 (35.1) |  |  | 1,301 (35.2) |  |  | 1,279 (35.2) |
| ≥ 12 |  | 1,134 (30.9) |  |  | 1,130 (30.5) |  |  | 1,127 (31.0) |
| Maternal skin color | 3,661 |  |  | 3,668 |  |  | 3,628 |  |
| White |  | 2,603 (71.0) |  |  | 2,616 (70.7) |  |  | 2,580 (71.0) |
| Black |  | 577 (15.8) |  |  | 594 (16.0) |  |  | 574 (15.8) |
| Brown or others |  | 481 (13.2) |  |  | 488 (13.3) |  |  | 474 (13.2) |
| Family income (quintiles) | 3,665 |  |  | 3,702 |  |  | 3,632 |  |
| Poorest |  | 710 (19.4) |  |  | 722 (19.5) |  |  | 696 (19.2) |
| Second |  | 722 (19.7) |  |  | 731 (19.7) |  |  | 710 (19.6) |
| Third |  | 749 (20.4) |  |  | 755 (20.4) |  |  | 746 (20.5) |
| Fourth |  | 761 (20.8) |  |  | 770 (20.8) |  |  | 760 (20.9) |
| Richest |  | 723 (19.7) |  |  | 724 (19.6) |  |  | 720 (19.8) |
| Mother living with a partner | 3,666 |  |  | 3,703 |  |  | 3,633 |  |
| Yes |  | 3,168 (86.4) |  |  | 3,201 (86.4) |  |  | 3,148 (86.6) |
| No |  | 498 (13.6) |  |  | 502 (13.6) |  |  | 485 (13.4) |
| Maternal occupation | 3,666 |  |  | 3,703 |  |  | 3,633 |  |
| Paid job |  | 2,085 (56.9) |  |  | 2,099 (56.7) |  |  | 2,705 (57.1) |
| Unpaid job |  | 1,581 (43.1) |  |  | 1,604 (43.3) |  |  | 1,558 (42.9) |
| Parity | 3,665 |  |  | 3,702 |  |  | 3,632 |  |
| 1 |  | 1,817 (49.6) |  |  | 1,836 (49.6) |  |  | 1,805 (49.7) |
| 2 |  | 1,146 (31.3) |  |  | 1,156 (31.2) |  |  | 1,138 (31.3) |
| ≥ 3 |  | 702 (19.1) |  |  | 710 (19.2) |  |  | 689 (19.0) |
| Smoking in pregnancy | 3,664 |  |  | 3,701 |  |  | 3,631 |  |
| Yes |  | 583 (15.9) |  |  | 592 (16.0) |  |  | 575 (15.8) |
| No |  | 3,081 (84.1) |  |  | 3,109 (84.0) |  |  | 3,056 (84.2) |
| Number of antenatal care visits | 3,602 |  |  | 3,651 |  |  | 3,585 |  |
| < 6 |  | 441 (12.2) |  |  | 463 (12.7) |  |  | 435 (12.2) |
| 6-8 |  | 1,300 (36.1) |  |  | 1,318 (36.1) |  |  | 1,294 (36.1) |
| ≥ 9 |  | 1,861 (51.7) |  |  | 1,870 (51.2) |  |  | 1,856 (51.7) |
| Type of delivery | 3,666 |  |  | 3,703 |  |  | 3,633 |  |
| Vaginal |  | 1,296 (35.3) |  |  | 1,323 (35.7) |  |  | 1,289 (35.3) |
| Cesarean section |  | 2.370 (64.7) |  |  | 2,380 (64.3) |  |  | 2,350 (64.7) |
| Birth weight (grams) | 3,664 |  |  | 3,701 |  |  | 3,631 |  |
| < 2,500 |  | 280 (7.7) |  |  | 283 (7.7) |  |  | 276 (7.6) |
| 2,500-3,500 |  | 2,379 (64.9) |  |  | 2,403 (64.9) |  |  | 2,363 (65.0) |
| ≥ 3500 |  | 1,005 (27.4) |  |  | 1,015 (27.4) |  |  | 992 (27.4) |
| Preterm birth (< 37 weeks gestation) | 3,666 |  |  | 3,703 |  |  | 3,633 |  |
| Yes |  | 478 (13.0) |  |  | 480 (13.0) |  |  | 473 (13.0) |
| No |  | 3,188 (87.0) |  |  | 3,223 (87.0) |  |  | 3,160 (87.0) |
| Sex of the child | 3,666 |  |  | 3,703 |  |  | 3,633 |  |
| Boy |  | 1,868 (50.9) |  |  | 1,880 (50.7) |  |  | 1,845 (50.8) |
| Girl |  | 1,798 (49.1) |  |  | 1,823 (49.3) |  |  | 1,788 (49.2) |

^a^Only singleton births^;^ ^b^Totals differ due to missing values; ^c^Significant differences were not observed for characteristics among all groups (P>0.05).

Table S3. Univariate analysis between maternal and child characteristics and suspected child development delay^a^ among boys in the 2015 Pelotas (Brazil) Birth Cohort (n = 1,924).

| Characteristics | n | Global | Language | Cognitive | Motor |
| --- | --- | --- | --- | --- | --- |
|  |  | OR (95% CI) | | | |
| Maternal age (years) | 1,924 |  |  |  |  |
| < 20 |  | 0.85 (0.56-1.30) | 0.89 (0.60-1.33) | 0.89 (0.60-1.31) | 0.93 (0.59-1.46) |
| 20-35 |  | Reference | Reference | Reference | Reference |
| ≥ 35 |  | 1.16 (0.79-1.69) | 1.08 (0.74-1.58) | 1.19 (0.83-1.69) | 1.14 (0.75-1.73) |
| Maternal schooling (years) | 1,923 |  |  |  |  |
| 0-4 |  | 0.74 (0.42-1.29) | 0.90 (0.53-1.52) | 1.13 (0.70-1.82) | 1.23 (0.70-2.13) |
| 5-8 |  | 0.93 (0.64-1.33) | 1.13 (0.79-1.60) | 1.17 (0.83-1.65) | 1.03 (0.68-1.56) |
| 9-11 |  | 0.95 (0.67-1.33) | 0.99 (0.71-1.39) | 1.04 (0.75-1.44) | 1.15 (0.79-1.68) |
| ≥ 12 |  | Reference | Reference | Reference | Reference |
| Maternal skin color | 1,923 |  |  |  |  |
| White |  | Reference | Reference | Reference | Reference |
| Black |  | 0.81 (0.54-1.23) | 0.89 (0.61-1.31) | 0.85 (0.59-1.24) | 0.76 (0.48-1.21) |
| Brown or other |  | 1.32 (0.91-1.92) | 0.91 (0.61-1.37) | 1.06 (0.73-1.54) | 1.23 (0.81-1.87) |
| Family income (quintiles) | 1,923 |  |  |  |  |
| Poorest |  | 1.22 (0.78-1.90) | 1.09 (0.71-1.67) | 1.20 (0.80-1.81) | 0.95 (0.58-1.52) |
| Second |  | 1.31 (0.83-2.04) | 1.25 (0.82-1.91) | 1.24 (0.82-1.87) | 1.10 (0.68-1.76) |
| Third |  | 1.12 (0.71-1.75) | 0.88 (0.57-1.37) | 0.97 (0.64-1.49) | 0.76 (0.46-1.25) |
| Fourth |  | 1.11 (0.71-1.74) | 1.04 (0.68-1.60) | 1.10 (0.73-1.67) | 0.98 (0.61-1.56) |
| Richest |  | Reference | Reference | Reference | Reference |
| Mother living with a partner | 1,923 |  |  |  |  |
| Yes |  | Reference | Reference | Reference | Reference |
| No |  | 1.07 (0.72-1.58) | 1.15 (0.79-1.67) | 0.96 (0.66-1.40) | 0.95 (0.61-1.48) |
| Maternal occupation | 1,924 |  |  |  |  |
| Paid job |  | Reference | Reference | Reference | Reference |
| Unpaid job |  | 1.08 (0.82-1.43) | 1.11 (0.84-1.45) | 1.14 (0.88-1.48) | 1.25 (0.92-1.70) |
| Parity | 1,923 |  |  |  |  |
| 1 |  | Reference | Reference | Reference | Reference |
| 2 |  | 0.88 (0.64-1.22) | 0.84 (0.60-1.16) | 0.90 (0.66-1.22) | 0.92 (0.64-1.30) |
| ≥ 3 |  | 1.11 (0.77-1.58) | 1.46 (1.04-2.04) | 1.34 (0.97-1.86) | 1.02 (0.68-1.53) |
| Smoking in pregnancy | 1,923 |  |  |  |  |
| Yes |  | 0.84 (0.57-1.24) | 1.11 (0.78-1.59) | 1.06 (0.75-1.49) | 1.13 (0.75-1.68) |
| No |  | Reference | Reference | Reference | Reference |
| Number of antenatal care visits | 1,878 |  |  |  |  |
| < 6 |  | 1.98 (1.35-2.93) | 1.97 (1.35-2.89) | 1.89 (1.30-2.73) | 1.69 (1.11-2.59) |
| 6-8 |  | 1.35 (0.98-1.84) | 1.35 (1.00-1.84) | 1.38 (1.04-1.85) | 1.11 (0.79-1.57) |
| ≥ 9 |  | Reference | Reference | Reference | Reference |
| Type of delivery | 1,923 |  |  |  |  |
| Vaginal |  | Reference | Reference | Reference | Reference |
| Cesarean section |  | 0.94 (0.71-1.26) | 1.05 (0.79-1.39) | 1.09 (0.83-1.43) | 0.97 (0.71-1.34) |
| Birth weight (grams) | 1,923 |  |  |  |  |
| < 2,500 |  | 1.55 (0.92-2.62) | 1.65 (1.01-2.67) | 1.61 (1.01-2.57) | 1.07 (0.52-1.95) |
| 2,500-3,500 |  | 1.20 (0.88-1.64) | 0.97 (0.72-1.31) | 0.99 (0.75-1.32) | 0.96 (0.69-1.33) |
| ≥ 3500 |  | Reference | Reference | Reference | Reference |
| Preterm birth (< 37 weeks gestation) | 1,924 |  |  |  |  |
| Yes |  | 2.10 (1.49-2.95) | 2.11 (1.52-2.95) | 2.03 (1.47-2.80) | 1.79 (1.22-2.62) |
| No |  | Reference | Reference | Reference | Reference |

^a^Suspected child development delay defined as scores of each domain below 10^th^ percentile, based on the entire 2015 Pelotas (Brazil) Birth Cohort.

OR – odds ratio; 95% CI – 95% confidence interval

Table S4. Univariate analysis between maternal and child characteristics and suspected child development delay^a^ among girls in the 2015 Pelotas (Brazil) Birth Cohort (n = 1,852).

| Characteristics | n | Global | Language | Cognitive | Motor |
| --- | --- | --- | --- | --- | --- |
|  |  | OR (95% CI) | | | |
| Maternal age (years) | 1,852 |  |  |  |  |
| < 20 |  | 0.98 (0.61-1.59) | 0.92 (0.54-1.56) | 1.12 (0.71-1.77) | 1.24 (0.82-1.87) |
| 20-35 |  | Reference | Reference | Reference | Reference |
| ≥ 35 |  | 0.96 (0.59-1.57) | 1.06 (0.64-1.76) | 1.21 (0.77-1.89) | 1.19 (0.78-1.81) |
| Maternal schooling (years) | 1,852 |  |  |  |  |
| 0-4 |  | 2.27 (1.24-4.16) | 1.81 (0.92-3.54) | 2.25 (1.25-4.06) | 1.44 (0.85-2.42) |
| 5-8 |  | 1.64 (1.01-2.66) | 1.79 (1.08-2.95) | 1.85 (1.17-2.93) | 0.90 (0.59-1.35) |
| 9-11 |  | 1.63 (1.03-2.57) | 1.49 (0.91-2.41) | 1.46 (0.93-2.29) | 0.92 (0.63-1.34) |
| ≥ 12 |  | Reference | Reference | Reference | Reference |
| Maternal skin color | 1,852 |  |  |  |  |
| White |  | Reference | Reference | Reference | Reference |
| Black |  | 0.84 (0.52-1.36) | 0.97 (0.60-1.57) | 0.80 (0.50-1.27) | 0.82 (0.53-1.27) |
| Brown or other |  | 0.89 (0.53-1.50) | 0.72 (0.39-1.31) | 0.70 (0.41-1.21) | 0.95 (0.60-1.50) |
| Family income (quintiles) | 1,851 |  |  |  |  |
| Poorest |  | 2.36 (1.26-4.40) | 1.38 (0.75-2.54) | 1.76 (1.00-3.07) | 1.35 (0.82-2.24) |
| Second |  | 2.85 (1.55-5.23) | 1.61 (0.90-2.90) | 1.90 (1.10-3.27) | 1.52 (0.93-2.48) |
| Third |  | 1.61 (0.83-3.12) | 1.10 (0.58-2.07) | 1.23 (0.68-2.22) | 1.26 (0.76-2.10) |
| Fourth |  | 2.04 (1.08-3.83) | 1.53 (0.85-2.77) | 1.48 (0.83-2.61) | 1.11 (0.66-1.86) |
| Richest |  | Reference | Reference | Reference | Reference |
| Mother living with a partner | 1,852 |  |  |  |  |
| Yes |  | Reference | Reference | Reference | Reference |
| No |  | 0.87 (0.53-1.44) | 0.83 (0.48-1.43) | 1.03 (0.65-1.64) | 1.07 (0.70-1.63) |
| Maternal occupation | 1,852 |  |  |  |  |
| Paid job |  | Reference | Reference | Reference | Reference |
| Unpaid job |  | 1,58 (1.13-2.21) | 1.27 (0.89-1.81) | 1.42 (1.03-1.97) | 1.62 (1.20-2.19) |
| Parity | 1,851 |  |  |  |  |
| 1 |  | Reference | Reference | Reference | Reference |
| 2 |  | 1.00 (0.67-1.50) | 1.21 (0.79-1.87) | 1.21 (0.81-1.79) | 0.82 (0.58-1.17) |
| ≥ 3 |  | 1.62 (1.08-2.43) | 2.06 (1.34-3.17) | 2.11 (1.42-3.13) | 0.86 (0.58-1.29) |
| Smoking in pregnancy | 1,850 |  |  |  |  |
| Yes |  | 1.51 (1.00-2.27) | 1.23 (0.78-1.94) | 1.28 (0.84-1.93) | 0.97 (0.64-1.46) |
| No |  | Reference | Reference | Reference | Reference |
| Number of antenatal care visits | 1,852 |  |  |  |  |
| < 6 |  | 1.23 (0.72-2.11) | 1.74 (1.04-2.94) | 1.86 (1.15-2.99) | 1.06 (0.66-1.70) |
| 6-8 |  | 1.57 (1.09-2.25) | 1.39 (0.93-2.07) | 1.54 (1.07-2.22) | 1.12 (0.81-1.56) |
| ≥ 9 |  | Reference | Reference | Reference | Reference |
| Type of delivery | 1,852 |  |  |  |  |
| Vaginal |  | Reference | Reference | Reference | Reference |
| Cesarean section |  | 0.83 (0.59-1.16) | 0.70 (0.49-1.01) | 0.66 (0.47-0.91) | 0.87 (0.64-1.19) |
| Birth weight (grams) | 1,852 |  |  |  |  |
| < 2,500 |  | 1.41 (0.78-2.55) | 2.10 (1.17-3.78) | 1.97 (1.13-3.45) | 1.42 (0.81-2.46) |
| 2,500-3,500 |  | 0.78 (0.52-1.16) | 0.82 (0.53-1.26) | 0.89 (0.59-1.33) | 0.89 (0.62-1.29) |
| ≥ 3500 |  | Reference | Reference | Reference | Reference |
| Preterm birth (< 37 weeks gestation) | 1,852 |  |  |  |  |
| Yes |  | 1.26 (0.79-2.01) | 1.68 (1.06-2.65) | 1.52 (0.98-2.34) | 1.65 (1.11-2.45) |
| No |  | Reference | Reference | Reference | Reference |

^a^Suspected child development delay defined as scores of each domain below 10^th^ percentile, based on the entire 2015 Pelotas (Brazil) Birth Cohort.

OR – odds ratio; 95% CI – 95% confidence interval

Table S5. Crude linear regression model between pre-pregnancy body mass index and total gestational weight gain with INTER-NDA score percentiles by domain at 24-month follow-up assessment in the 2015 Pelotas (Brazil) Birth Cohort, stratified by child’s sex.

|  | Global | Language | Cognitive | Motor |
| --- | --- | --- | --- | --- |
|  | β (95% CI) | β (95% CI) | β (95% CI) | β (95% CI) |
|  | Total sample | | | |
| Pre-pregnancy BMI (kg/m^2^)^a^ | -0.002 (-0.008; 0.003) | -0.002 (-0.008; 0.004) | -0.000 (-0.006; 0.005) | 0.003 (-0.002; 0.009) |
| Total gestational weight gain (kg)^b^ | 0.001 (-0.003; 0.006) | 0.003 (-0.001; 0.008) | 0.003 (-0.001; 0.008) | -0.002 (-0.007; 0.002) |
|  | Boys | | | |
| Pre-pregnancy BMI (kg/m^2^)^a^ | -0.001 (-0.009; 0.006) | 0.000 (-0.008; 0.008) | 0.001 (-0.006; 0.009) | 0.000 (-0.007; 0.008) |
| Total gestational weight gain (kg)^b^ | -0.003 (-0.010; 0.003) | -0.002 (-0.009; 0.004) | -0.002 (-0.009; 0.004) | -0.004 (-0.011; 0.002) |
|  | Girls | | | |
| Pre-pregnancy BMI (kg/m^2^)^a^ | -0.002 (-0.011; 0.006) | -0.002 (-0.010; 0.006) | -0.000 (-0.009; 0.007) | 0.005 (-0.003; 0.014) |
| Total gestational weight gain (kg)^b^ | 0.006 (-0.002; 0.013) | 0.010 (0.003; 0.016) | 0.009 (0.002; 0.016) | -0.000 (-0.007; 0.006) |

INTER-NDA – Intergrowth-21^st^ Neurodevelopment Assessment. BMI – body mass index. 95% CI - 95% confidence interval.

^a^n = 3,666; ^b^n = 3,703

Table S6. Crude association between pre-pregnancy body mass index and adherence to IOM total gestational weight gain recommendations with suspected child development delay^a^ at 24-months, in the total sample, and stratified by child sex, in the 2015 Pelotas (Brazil) Birth Cohort (n = 3,913).

|  | Global | Language | Cognitive | Motor |
| --- | --- | --- | --- | --- |
|  | OR (95% CI) | OR (95% CI) | OR (95% CI) | OR (95% CI) |
|  | Total sample | | | |
| Pre-pregnancy BMI (kg/m^2^) |  |  |  |  |
| < 18.5 | 1.30 (0.77-2.19) | 1.09 (0.62-1.90) | 0.96 (0.56-1.65) | 1.48 (0.90-2.44) |
| 18.5-24.9 | Reference | Reference | Reference | Reference |
| 25.0-29.9 | 1.08 (0.84-1.39) | 1.09 (0.84-1.40) | 1.02 (0.80-1.30) | 1.05 (0.81-1.36) |
| ≥ 30.0 | 0.94 (0.70-1.26) | 1.01 (0.75-1.36) | 0.91 (0.69-1.21) | 0.90 (0.66-1.22) |
| Total gestational weight gain (kg)^b^ |  |  |  |  |
| Insufficient | 0.90 (0.68-1.18) | 1.06 (0.80-1.40) | 1.10 (0.85-1.43) | 0.76 (0.58-1.01) |
| Adequate | Reference | Reference | Reference | Reference |
| Excessive | 1.01 (0.78-1.31) | 1.20 (0.92-1.56) | 1.20 (0.93-1.54) | 0.98 (0.76-1.27) |
|  | Boys | | | |
| Pre-pregnancy BMI (kg/m^2^) |  |  |  |  |
| < 18.5 | 0.74 (0.33-1.67) | 0.47 (0.18-1.19) | 0.50 (0.21-1.18) | 0.82 (0.34-1.94) |
| 18.5-24.9 | Reference | Reference | Reference | Reference |
| 25.0-29.9 | 0.97 (0.70-1.36) | 0.96 (0.69-1.32) | 0.95 (0.70-1.29) | 0.91 (0.63-1.33) |
| ≥ 30.0 | 0.81 (0.55-1.19) | 0.80 (0.55-1.16) | 0.74 (0.51-1.05) | 0.97 (0.65-1.46) |
| Total gestational weight gain (kg)^b^ |  |  |  |  |
| Insufficient | 0.91 (0.63-1.32) | 1.16 (0.81-1.67) | 1.26 (0.89-1.77) | 0.73 (0.48-1.11) |
| Adequate | Reference | Reference | Reference | Reference |
| Excessive | 1.18 (0.84-1.65) | 1.50 (1.07-2.10) | 1.49 (1.08-2.06) | 1.25 (0.87-1.79) |
|  | Girls | | | |
| Pre-pregnancy BMI (kg/m^2^) |  |  |  |  |
| < 18.5 | 2.31 (1.16-4.06) | 2.60 (1.26-5.36) | 1.91 (0.93-3.89) | 2.27 (1.21-4.23) |
| 18.5-24.9 | Reference | Reference | Reference | Reference |
| 25.0-29.9 | 1.23 (0.83-1.83) | 1.32 (0.86-2.04) | 1.11 (0.75-1.65) | 1.20 (0.85-1.71) |
| ≥ 30.0 | 1.10 (0.69-1.77) | 1.39 (0.85-2.26) | 1.19 (0.76-1.87) | 0.81 (0.51-1.28) |
| Total gestational weight gain (kg)^b^ |  |  |  |  |
| Insufficient | 0.89 (0.59-1.34) | 0.93 (0.60-1.44) | 0.93 (0.62-1.39) | 0.78 (0.54-1.14) |
| Adequate | Reference | Reference | Reference | Reference |
| Excessive | 0.78 (0.51-1.18) | 0.79 (0.51-1.23) | 0.84 (0.56-1.26) | 0.76 (0.53-1.10) |

OR -odds ratio. 95% CI - 95% confidence interval.

^a^Suspected child development delay defined as scores of each domain below 10^th^ percentile, based on the entire 2015 Pelotas (Brazil) Birth Cohort.

^b^According to Institute of Medicine guidelines, 2009^5^.

Table S7. Estimates of the mediated effect of total gestational weight gain^a^ on the association between pre-pregnancy body mass index^b^ with INTER-NDA score percentiles by domain in the 2015 Pelotas (Brazil) Birth Cohort (n = 3,633).

|  |  | Child development at 24 months of age | | |
| --- | --- | --- | --- | --- |
|  |  | Entire sample | Boys | Girls |
|  |  | β (95% CI) | β (95% CI) | β (95% CI) |
|  | TCE | -0.024 (-0.065; 0.019) | 0.008 (-0.052; 0.070) | 0.012 (-0.049; 0.075) |
| Global | NDE | 0.033 (-0.010; 0.077) | 0.004 (-0.057; 0.066) | -0.011 (-0.074; 0.051) |
|  | NIE | -0.057 (-0.101; -0.013) | 0.004 (-0.056; 0.065) | 0.024 (-0.038; 0.086) |
|  | TCE | -0.010 (-0.053; 0.032) | 0.012 (-0.048; 0.072) | -0.048 (-0.109; 0.011) |
| Language | NDE | -0.010 (-0.045; 0.041) | -0.012 (-0.074; 0.049) | -0.034 (-0.094; 0.025) |
|  | NIE | -0.008 (-0.052; 0.034) | 0.024 (-0.036; 0.085) | -0.014 (-0.073; 0.044) |
|  | TCE | -0.022 (-0.065; 0.020) | 0.008 (-0.052; 0.068) | -0.007 (-0.068; 0.053) |
| Cognitive | NDE | 0.017 (-0.026; 0.060) | -0.002 (-0.064; 0.059) | 0.000 (-0.060; 0.061) |
|  | NIE | -0.039 (-0.082; 0.003) | 0.010 (-0.049; 0.070) | -0.007 (-0.067; 0.051) |
|  | TCE | 0.007 (-0.036; 0.051) | -0.053 (-0.115; 0.007) | 0.042 (-0.020; 0.106) |
| Motor | NDE | 0.025 (-0.018; 0.069) | -0.026 (-0.087; 0.035) | 0.045 (-0.017; 0.107) |
|  | NIE | -0.017 (-0.061; 0.026) | -0.027 (-0.087; 0.032) | -0.002 (-0.065; 0.060) |

^a^Gestational weight gain in kg.

^b^Body mass index in kg/m^2^.

TCE - estimates of the total effect of pre-pregnancy body mass index through different pathways on percentiles of child development; NDE - estimates of the direct effect of pre-pregnancy body mass index on percentiles of child development; NIE - estimates of the indirect effects of pre-pregnancy body mass index mediated through total gestational weight gain on percentiles of child development.
